# X-ray-induced acoustic computed tomography and its applications in biomedicine

**DOI:** 10.1117/1.JBO.29.S1.S11510

**Published:** 2023-12-22

**Authors:** Yuchen Yan, Shawn (Liangzhong) Xiang

**Affiliations:** a University of California, Irvine, Department of Biomedical Engineering, Irvine, California, United States; b University of California, Irvine, Department of Radiological Sciences, Irvine, California, United States; c University of California, Irvine, Beckman Laser Institute & Medical Clinic, Irvine, California, United States

**Keywords:** X-ray-induced acoustic computed tomography, radiological imaging, radiation dosimetry, radiotherapy monitoring, X-ray, acoustics

## Abstract

**Significance:**

X-ray-induced acoustic computed tomography (XACT) offers a promising approach to biomedical imaging, leveraging X-ray absorption contrast. It overcomes the shortages of traditional X-ray, allowing for more advanced medical imaging.

**Aim:**

The review focuses on the significance and draws onto the potential applications of XACT to demonstrate it as an innovative imaging technique

**Approach:**

This review navigates the expanding landscape of XACT imaging within the biomedical sphere. Integral topics addressed encompass the refinement of imaging systems and the advancement in image reconstruction algorithms. The review particularly emphasizes XACT’s significant biomedical applications.

**Results:**

Key uses, such as breast imaging, bone density maps for osteoporosis, and X-ray molecular imaging, are highlighted to demonstrate the capability of XACT. A unique niche for XACT imaging is its application in *in vivo* dosimetry during radiotherapy, which has been validated on patients.

**Conclusions:**

Because of its unique property, XACT has great potential in biomedicine and non-destructive testing. We conclude by casting light on potential future avenues in this promising domain.

## 1 Introduction

X-ray imaging has limitations that have persisted for over a century, including the requirement for the X-ray detector to be placed opposite the source and the risk of ionizing radiation as a carcinogen.^1^^,^^2^ X-ray-induced acoustic computed tomography (XACT) is a novel modality that has emerged in recent years to overcome these limitations.^2^^,^^3^ XACT uses short-pulsed X-ray beams to generate acoustic signals that can be used for tomographic imaging, with a linear relationship between dose and the X-ray-induced acoustic (XA) signal established. This opens up the possibility for XACT to be used in radiation therapy monitoring.^4^^,^^5^ XACT also has potential applications in both non-destructive testing^6^ and medicine.^4^^,^^5^^,^^7^^–^^13^

XACT imaging’s development is linked to important moments in the history of X-ray imaging. It emerged after computed tomography (CT) and the discovery of ultrasound caused by X-ray in 1983.^14^ In 1991, a key step happened when acoustic emissions were noticed. By placing a device perpendicular to a carefully aimed X-ray beam under water, a clear XA signal emerged.^12^ It was expected to be more pronounced in soft tissues due to their higher thermal expansion coefficient. In 2013, a major breakthrough occurred. The first X-ray acoustic computed tomography image was revealed. It displayed a two-dimensional (2D) picture of a lead rod inside a chicken breast, viewed from different angles using X-rays from a medical linear accelerator (LINAC).^5^ Notably, a direct link was seen between XA output and dosage, opening doors for monitoring therapy.^15^

## 2 Theory

The fundamental principle of XA phenomenon is the X-ray absorption of electrons that causes thermal increases in the scale of Milliken and generates pressure waves of XA signals.^16^ The XA effect can be described by the following equation:^17^

$$(\nabla^{2}-\frac{1}{v_{s}^{2}}\frac{\partial^{2}}{\partial t^{2}})p(\overset{\to }{r},t)=-\frac{\beta }{C_{p}}\frac{\partial H(\overset{\to }{r},t)}{\partial t},$$ (1)

where $v_{s}$ is the speed of sound, $p(\vec{r},t)$ is the acoustic pressure at location $\overset{\to }{r}$ with time t, β is thermal coefficient, $C_{p}$ represents the heat capacity under constant pressure, and $H(\vec{r},t)$ denotes to the heat function at location $\vec{r}$ and time t. For a short X-ray pulse, the local initial pressure rise $p_{0}$ can be written as

$$p_{0}=\Gamma \times \eta_{\mathrm{th}}\times \mu \times F,$$ (2)

where Γ denotes to dimensionless Gruneisen parameter $\Gamma =\beta v_{s}^{2}/C_{p}$, thermal efficiency $\eta_{\mathrm{th}}$, X-ray absorption coefficient μ, and F is the X-ray fluence.^18^ From Eq. (2), the XA signal is linearly related to the X-ray absorption; therefore, it leads to dosimetry measurement capability of XACT.^5^ Further expanding $\mu =\sigma \rho N_{A}/A$, where σ is the absorption cross section, ρ is the mass density, $N_{A}$ is the Avogadro number, and A is atomic number. Changes in density of the object or the X-ray absorption lead to change in XA signal proportionally, the biomedical imaging is achievable.^3^^,^^19^

## 3 Technology Development

**Fig. 1:**
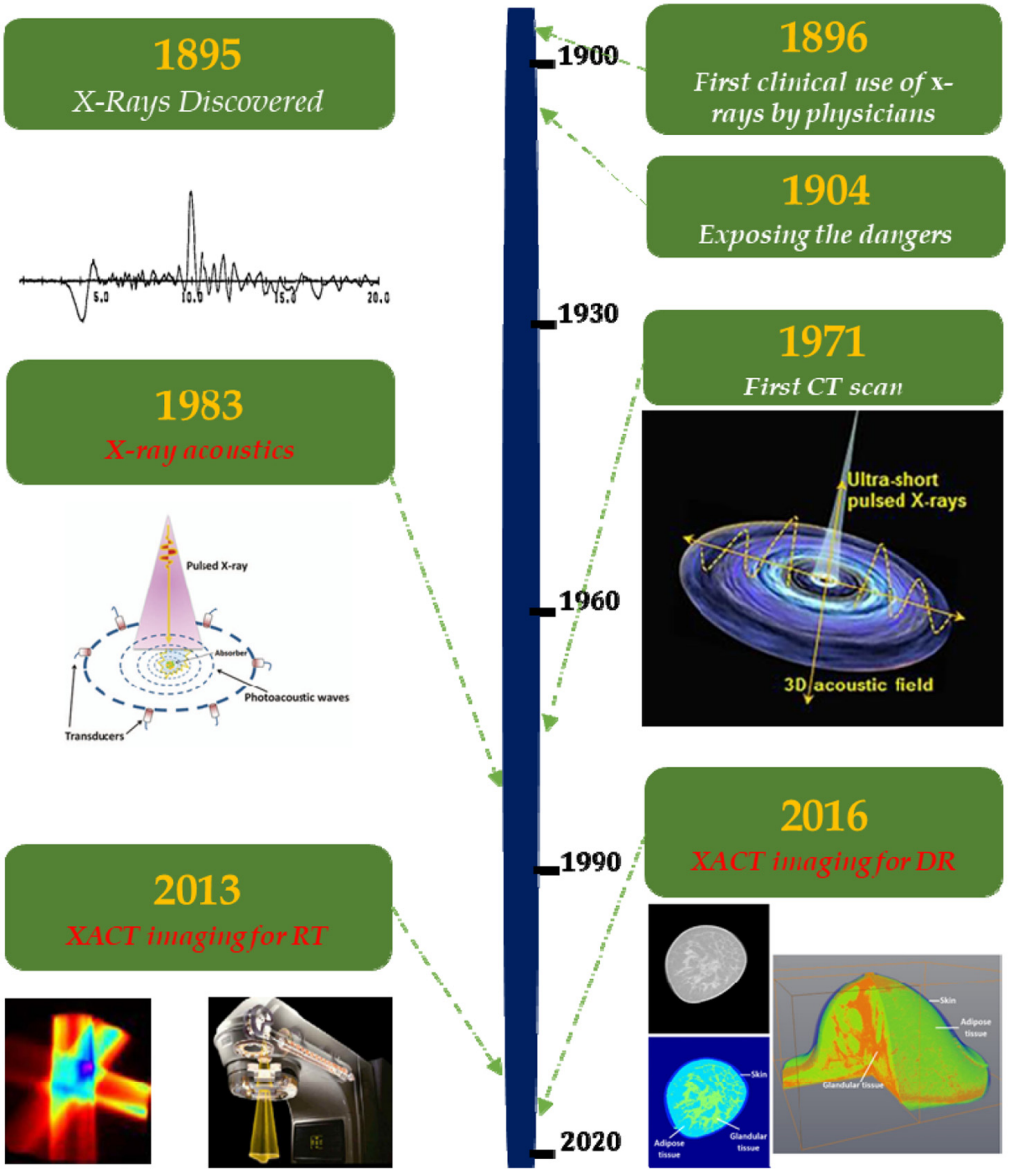
Timeline of XACT imaging in the history of X-ray imaging. In 1895, the first X-ray has been discovered. One year later, X-ray has been first applied to clinical use. In 1904, the dangers of X-rays were discovered during Edison’s research. In 1971, the first CT-Scan was taken by Dr. Ambrose. Reproduced with permission from Ref. 3. In 1983, X-ray acoustic effect has been discovered. Reproduced with permission from Ref. 5. In 2013, XACT was first introduced, and its application in imaging radiation therapy with a LINAC was found. Reproduced with permission from Ref. 20. In 2016, XACT has been found useful for diagnostic imaging. Reproduced with permission from Ref. 21.

The development of XACT imaging technology can be categorized into two main areas. The first aspect involves enhancing imaging instruments, which encompasses the utilization of various ultrasound transducers and arrays with different configurations. Notably, advancements in dedicated pre-amplifiers and data acquisition systems have been realized in recent years, leading to improved imaging sensitivity. The second facet pertains to software development, primarily focusing on refining signal processing and image reconstruction algorithms. In Secs. 3.1–3.3, we delve into the majority of the recent advancements in these domains (Fig. 1).

### 3.1 Instrumentation

**Fig. 2:**
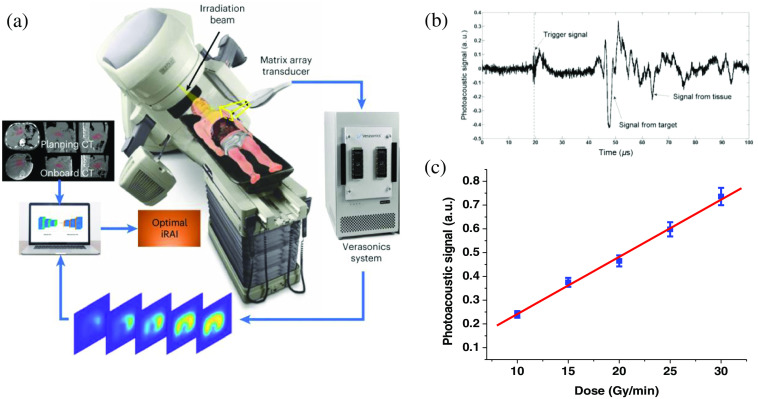
Rapid evolution of XACT imaging: from conceptual idea to clinical testing on patients. (a) 3D demonstration of radiation acoustic imaging system, showing the capability of radiation acoustic imaging in real-time clinical radiotherapy treatment monitoring. Reproduced with permission from Ref. 10. (b) The XACT signal measured by the transducer produced by X-ray absorption. Reproduced with permission from Ref. 5. (c) A linear relationship was observed between XACT signal amplitude and dose deposition, lay the foundation for its application in dosimetry. Reproduced with permission from Ref. 5.

In the early stages of XACT, single-element ultrasound transducers took center stage. These transducers could detect a single A-line signal^3^^,^^5^^,^^12^^,^^14^ while achieving 2D imaging involved mechanically rotating the detector across various angles, typically spanning 360 deg with 120 steps.^2^ Subsequently, ring array transducers with 128 elements arranged in a circular configuration emerged for comprehensive panoramic XACT imaging.^22^^,^^23^ Additionally, linear arrays, commonly used in clinical ultrasound applications, were integrated into experiments.^6^ As XACT imaging began to offer additional soft tissue morphological insights, the natural progression led to incorporating pulse and echo capabilities^7^ (Fig. 2).

To achieve three-dimensional (3D) imaging in XACT, matrix arrays became essential.^2^ Alternatively, rotating a curved array also unlocked the potential for 3D imaging.^24^ In essence, the instrumentation evolution for XACT imaging aligns with the principles of laser-induced photoacoustic imaging (PAI),^10^ and traditional pulse-echo ultrasound imaging, all while accommodating the specific need for lower ultrasound frequencies.

Moreover, the pivotal role of dedicated pre-amplifiers and data acquisition systems cannot be overstated in XACT imaging.^22^ Given the relatively weak nature of XA signals, substantial dB amplification is imperative to enhance signal amplitude for effective detection.^22^^,^^25^

### 3.2 Image Reconstruction Algorithm

Turning to the software aspect of XACT imaging development, it can be divided into two main components: signal processing and image reconstruction. Given the relatively modest strength of XA signals and the typically not very high signal-to-noise ratio (SNR) in raw data, various strategies have been explored to enhance this ratio. For example, wavelet filtering has been implemented to refine XA signals through decomposing the signal using wavelet transform and filter the signal in wavelet space then do the inversion to reconstruct the original signal.^26^ Signal averaging has been streamlined from thousands of instances to tens, or even eliminated. The trending field of deep learning also offers potential for noise reduction. Notably, the U-Net architecture has been utilized to boost the SNR using pre-trained data.^27^

**Fig. 3:**
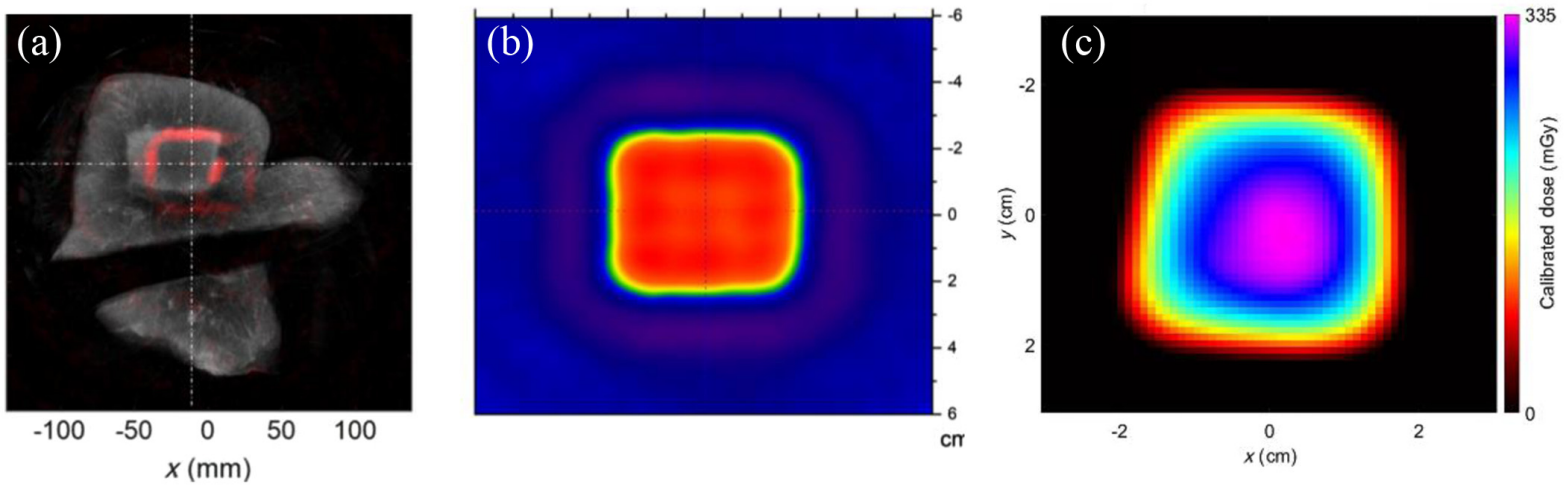
XACT imaging: bridging from beam tracking to quantitative dosimetry. (a) Ultrasound image showing the X-ray deposited (red rectangle) in a piece of veal liver phantom, proving the feasibility of obtaining beam alignment during radiotherapy. Reproduced with permission from Ref. 36. (b) XACT dose image with improved sensitivity showing the dose distribution of a single pulse; background are artifacts demonstrating a relative low SNR. Reproduced with permission from Ref. 4. (c) A quantitative measurement of the XACT does image is shown with all the artifacts being removed using a different algorithm. Reproduced with permission from Ref. 33.

When addressing cleaned XA signals, the challenge arises of translating these A-line signals back into an image. While many efforts have employed the straightforward back projection algorithm^3^^,^^28^ for image reconstruction [Fig. 3(b)], its accuracy faces limitations, particularly in scenarios with restricted views—a common occurrence in XACT imaging settings.^29^ To confront these limitations, model-based imaging reconstruction algorithms for XACT have been developed.^29^^–^^32^ The model-based reconstruction algorithm is a computationally demanding one that depends on the accurate calculation of the forward model of X-ray energy deposition with the implementation of a model matrix.^30^ The least-squares QR factorization method is also implemented to address the issue of insufficient storage when achieving a high data sampling rate, a large region of interest, and high resolution. Such intensive computation gives superiority to the model-based approach, which focuses on reducing noisy artifacts and allows for heterogeneous sound speed distribution.^29^^,^^30^ While they may not completely resolve the issue of limited views, these algorithms furnish more quantitative data [Fig. 3(c)],^33^ which holds significance, particularly for radiation dose monitoring. Subsequently, the model-based XACT algorithm has been extended to three dimensions.^29^ Given the vast amounts of calculations required for 3D datasets, a more efficient algorithm is needed. In recent decades, the rapid development of graphics processing units (GPUs) and Compute Unified Device Architecture by Nvidia Corporation has expanded the capabilities of GPU acceleration. GPU model-based reconstruction is expected to enhance performance and ideally achieve real-time reconstruction.^25^^,^^31^ Unquestionably, the deep learning is poised to wield substantial influence in image reconstruction, both in 2D and 3D contexts.^27^^,^^34^^,^^35^ With the help of deep learning algorithms, XACT reconstruction could be achieved with much less pulses as it increases the SNR. Also, it is expected that deep learning could be very useful in reducing the artifacts caused by limited views.^27^

### 3.3 Imaging Targets Usage

In the early stages of XACT, lead bars or rods were the primary imaging targets due to their high absorption of X-rays. These lead rods were typically embedded in agar phantoms or tissues, such as chicken breasts.^5^ Other metals, such as gold fiducial markers,^3^ have also been utilized in XACT. As the technology has developed, more experiments have focused on *in vitro* studies using liver tissues,^36^ mouse stomach,^37^ and bone samples^8^^,^^13^ embedded within agar phantoms. 3D digital phantoms have also been employed to demonstrate XACT’s capabilities in breast imaging.^15^^,^^21^ In the future, XACT is anticipated to further enhance imaging of soft tissue and needle placement during the interventional process.^38^

## 4 Applications

**Fig. 4:**
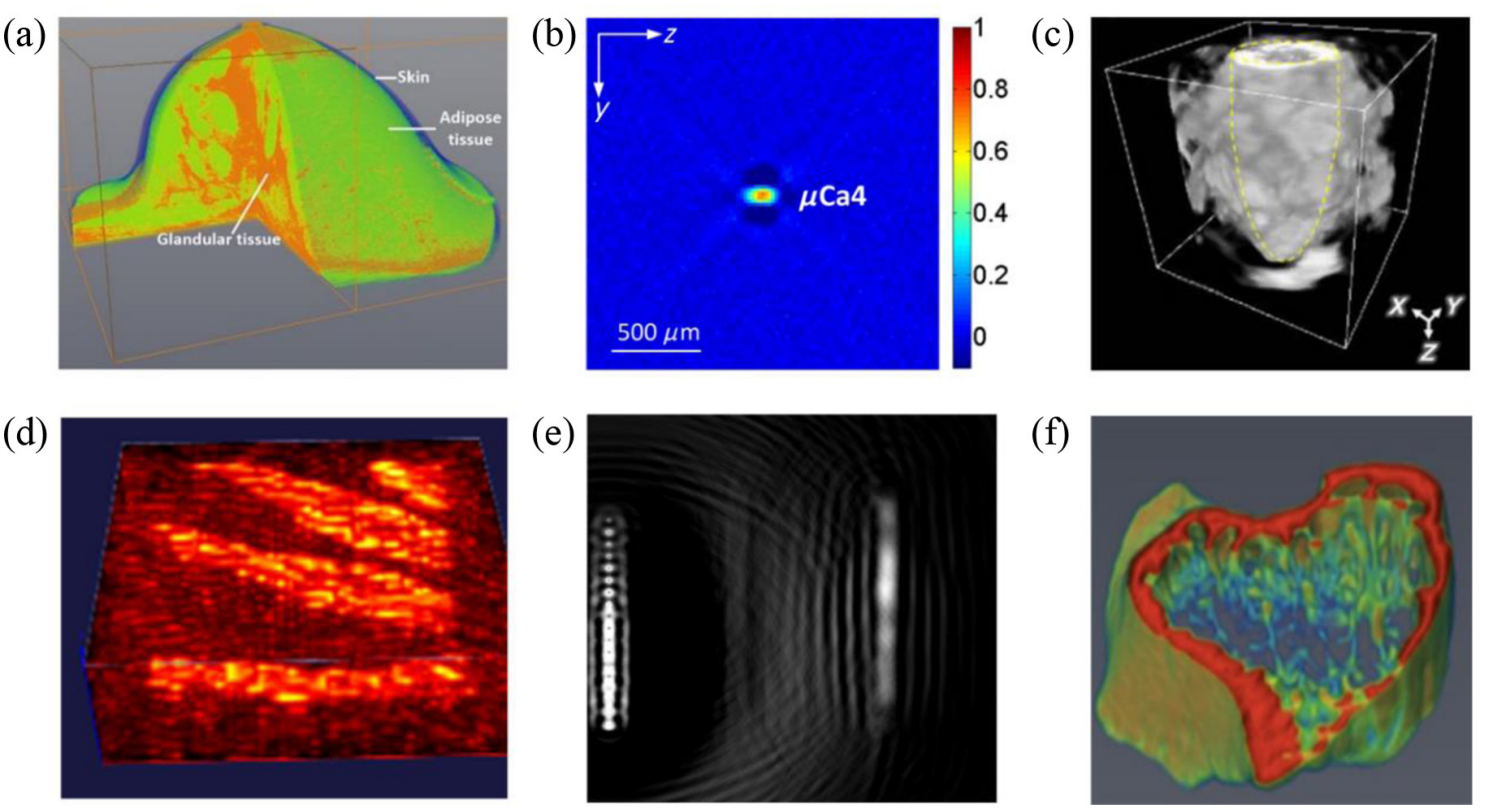
XACT and its application in radiological imaging. (a) 3D breast XACT image. Reproduced with permission from Ref. 21 (b) XACT images of the μCa cluster embedded into a breast. Reproduced with permission from Ref. 21. (c) A 3D volumetric XACT image of contrast agent within a chicken tissue. Reproduced with permission from Ref. 37. (d) 3D XACT image of a mouse paw showing the bone density distribution. Reproduced with permission from Ref. 13. (e) First ever XACT images of a chicken bone. Reproduced with permission from Ref. 8. (f) 3D XACT volumetric image of simulated osteoporotic bones. Reproduced with permission from Ref. 9.

XACT imaging and laser-induced PAI share similar physical principles, fostering a mutual opportunity for technological cross-pollination.^17^^,^^18^^,^^39^ However, a key divergence aphorizes in their distinct clinical applications. Sections 4.1 and 4.2 explore the potential clinical roles of XACT imaging (Fig. 4).

### 4.1 Radiological Imaging

Given X-ray’s historical precedence in radiological imaging, XACT imaging emerges with significant diagnostic promise. In recent years, the exploration of XACT imaging has predominantly centered around applications, such as breast imaging,^15^^,^^21^ bone density mapping,^8^^,^^9^^,^^13^ and the intriguing potential for molecular imaging using X-rays.^37^

#### 4.1.1 Breast imaging

Emerging as an innovative imaging technique, XACT demonstrates its capacity to image the breast, akin to dedicated breast CT or tomosynthesis, and holds advantages over the widely adopted gold standard in clinical settings—mammography. Through the utilization of a 3D breast digital phantom, simulation studies have been conducted, revealing that XACT imaging, when harnessed with a 7.5 MHz frequency transducer, achieves remarkable 100  μm resolution with a robust SNR.^21^ Furthermore, XACT effectively resolves the issue of tissue superposition that plagues traditional 2D mammography by employing 3D imaging. Leveraging the 3D propagation of XA waves, XACT facilitates comprehensive breast imaging while utilizing significantly reduced dosage (just 1/10 of the exposure from two-view mammography), thus mitigating potential health hazards associated with radiation overexposure.^15^^,^^21^

#### 4.1.2 Bone density map

XACT also offers distinct advantages over dual-energy X-ray absorptiometry and micro-CT for bone imaging. The study’s initial focus was on optimizing the configuration of the XACT imaging system tailored for bone imaging through computer simulations. The optimization process involved theoretical calculations and simulations on a mouse-sized digital phantom containing varying X-ray absorption coefficients. A reconstruction algorithm based on total variation was then utilized to reconstruct 3D XACT images from this refined setup.^13^

Subsequent experimentation was carried out on a piece of chicken bone, resulting in the first-ever experimental XACT image of a biological sample.^8^ Furthermore, XACT imaging was proposed as a means of visualizing the inner bone’s microarchitecture, yielding valuable insights into osteoporosis.^9^ As a result, XACT emerges as a promising imaging technique for detecting osteoporosis.

However, improving resolution remains a challenge, given the need for higher frequency ultrasound transducers. Yet, such frequencies often encounter significant attenuation within the bone, leading to substantial signal reflection.^11^ Looking ahead, real-world experiments involving actual bone specimens are anticipated to further underscore XACT’s potential in early osteoporosis detection and prevention. This method delivers 3D images encompassing both density and mechanical information, all while minimizing the applied dosage.

#### 4.1.3 Molecular imaging

In the realm of imaging, X-ray techniques have traditionally been distinguished from molecular imaging modalities. However, XACT introduces a new dimension by utilizing contrast agents to enable *in situ* imaging. An intriguing development has emerged where researchers have successfully harnessed XACT to capture 3D images of a mouse’s stomach.^37^ This achievement was facilitated by the administration of a conventional oral contrast agent known as Gastrografin. The resulting 3D rendered XACT image provides a compelling visual representation of how the contrast agent thoroughly fills the stomach within the mouse.^3^ This real-time visualization capability holds tremendous potential for understanding anatomical dynamics and processes within living organisms. Moreover, the integration of a contrast agent offers another significant benefit—a boost in the SNR of XACT imaging. In fact, this enhancement amounted to a notable 13% increase compared to using water alone as the imaging medium. This improvement in SNR directly translates to clearer and more discernible images, enriching the quality of the captured data.^37^ Furthermore, there is exciting potential on the horizon to elevate XACT imaging even further. By considering advanced array configurations, such as spherical or matrix arrays, the benefits of contrast agents could be maximized.^40^ Such arrays promise to amplify the SNR gains, potentially unlocking new realms of clarity and precision in XACT imaging.

In essence, the marriage of XACT imaging with contrast agents represents a groundbreaking stride in the realm of medical imaging. It introduces the capacity for *in situ* visualization and offers an enhanced understanding of biological processes at a molecular level, blurring the boundaries between conventional X-ray imaging and molecular imaging techniques.^37^^,^^40^

### 4.2 Radiotherapy Monitoring

**Fig. 5:**
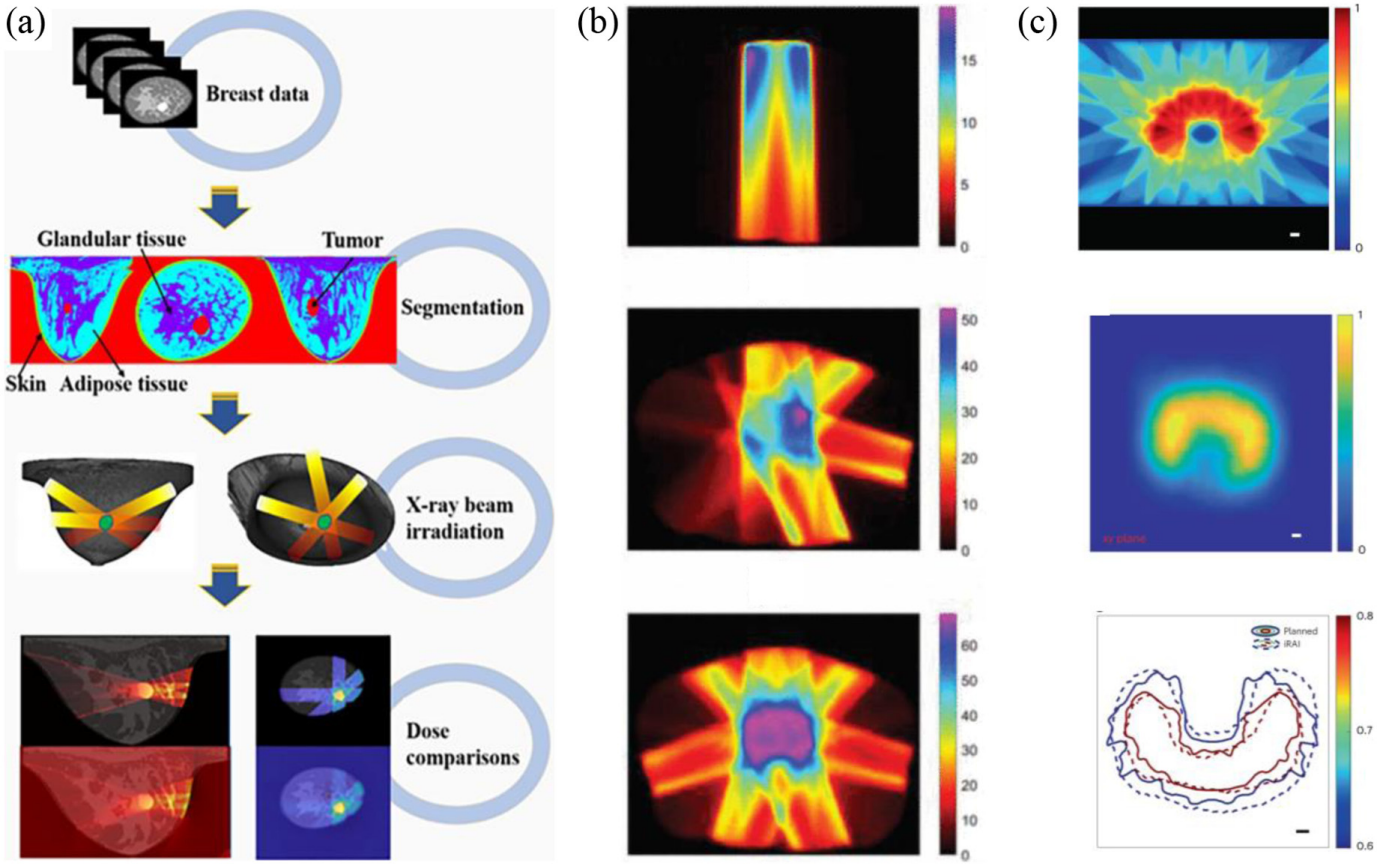
XACT and its application radiotherapy monitoring. (a) How to get XACT images for in vivo dosimetry, for example during partial breast irradiation. The first step is acquiring multiple sets of breast CT images; then generates/reconstructs the XACT image with the acquired XACT data; performs the image fusion between CT and XACT. Reproduced with permission from Ref. 15. (b) Dose distribution with one beam (top), four beams (middle), and seven beams (bottom) showing its real-time monitoring capability of dose deposition. Reproduced with permission from Ref. 20. (c) The first XACT image of dose distribution and accumulation in a real tissue-mimicking phantom. A treatment plan with dose distribution (top) is compared with XACT measurement of the dose distribution (middle) shows that XACT is very stable in monitoring radiotherapy process (bottom). Reproduced with permission from Ref. 10.

Among the myriad applications of XACT imaging, one stands out as particularly thrilling: its role in radiotherapy monitoring. This application has witnessed rapid development, evolving swiftly since its initial introduction for radiation therapy monitoring. What was once an innovative concept has now advanced to the stage of clinical testing, where XACT’s prowess in monitoring dose deposition during radiotherapy procedures has been validated (Fig. 5).

#### 4.2.1 Radiation beam visualization

The foundation for this monitoring capability lies in the proportional relationship between the intensity of the XA signal and the radiation dose deposition. This crucial connection empowers XACT to effectively monitor radiotherapy. The exploration has been most pronounced in liver^10^ and prostate cancer^20^^–^^42^ therapies, where XACT’s potential has been extensively investigated.

Lei et al.^36^ presented compelling evidence of XACT’s prowess by imaging phantoms containing veal livers and fat, using an X-ray beam on the target area. This demonstrated XACT’s ability to capture the position and distribution of radiation dose, holding promise for image-guided radiation therapy. In parallel, K-wave simulation studies have shed light on the potential for *in vivo* dosimetry of the prostate. By utilizing a transperineal 2D matrix array and analyzing varying radiation beam deliveries to prostates, the high dose distribution within the prostate area, juxtaposed with the sparing of the surrounding region, became evident.^20^

While numerous studies have hinged on simulation or *in vitro* experimentation, Zhang et al.^10^ embarked on an *in vivo* exploration using a rabbit model. Employing a dual-modality system, they achieved real-time tracking of the X-ray beam treatment on the target tissue.^10^ Nonetheless, it is imperative that more comprehensive patient studies ensue to definitively establish XACT’s prowess in monitoring real patients’ radiotherapy procedures.

The trajectory of XACT imaging in the domain of radiotherapy monitoring holds great promise, offering the potential to revolutionize how radiation doses are managed and guided, ultimately optimizing treatment outcomes for patients.

#### 4.2.2 *In vivo* dosimetry

While visualizing invisible radiation beam is significant, an even more crucial endeavor lies in quantifying the radiation dose in vivo for patients—a dimension currently absents from prevailing clinical practices. To this end, initial strides were made using X-ray emissions from a LINAC, generating images with a minimal dosage of 30  Gy/min. Particularly noteworthy is the identification of a linear correlation between X-ray acoustic output and dosage. This breakthrough discovery kindles the potential of XACT in the realm of therapeutic monitoring.^5^

This promising trajectory has spurred the development of novel imaging instrumentation and advanced image algorithms aimed at extracting precise quantitative dose information during radiotherapy. By harnessing the capabilities of XACT,^33^ clinicians and researchers are poised to usher in a new era of radiation therapy that not only visualizes but quantifies the therapeutic impact, culminating in more informed and optimized treatment strategies.

## 5 Outlook and Discussion

Inspired by Dr. Lihong Wang’s research in laser-induced PAI from decades ago,^43^ we suggested the use of X-rays to create acoustic waves for imaging in 2013. The research in PAI also motivates and contributes to the development of XACT. Reconstruction algorithms such as the conventional back projection^28^ and model-based^30^^,^^31^^,^^44^ in PAI have been adapted to XACT and further improved XACT’s reconstruction for getting quantitative information.^33^^,^^45^ The application of XACT imaging within the realm of biomedicine holds tremendous potential. The inherent connection between the XA signal and absorber density opens a captivating pathway—the ability to quantify human body density within images. This breakthrough concept presents a wide array of possibilities, akin to the dual-energy CT technique employed for bone densitometry.^8^^,^^9^^,^^13^ These advancements carry substantial biomedical implications, spanning from mapping bone density to advancing breast imaging techniques.^15^^,^^21^

Crucially, the amplitude of XA signals is intricately tied to the total X-ray dose absorbed by the patient’s body. This distinctive attribute takes a prominent role in clinical radiation therapy, offering a promising route to monitor and visualize dose distributions within targeted organs. By leveraging the proportional link between dose and acoustic signal, a pressure map emerges, shedding light on the relative dose distribution within tissue. This map acts as a valuable tool for cross-referencing with the precise location of the target organ, ensuring meticulous beam alignment and accurate dose administration.^15^^,^^33^^,^^36^^,^^20^^,^^46^ This serves as the foundation for XACT imaging’s potential in image-guided radiation therapy. A noteworthy advantage lies in XACT’s non-interference with the radiation beam; transducers positioned outside the beam path eliminate the need for correction factors prevalent in other dosimetry techniques.^20^

Despite these advancements, the journey toward achieving quantitative imaging through XACT remains a challenge, paralleling the trajectory observed in certain implementations of PAI. This underscores the ongoing importance of refining quantitative image reconstruction algorithms, a crucial endeavor for future applications.

XACT is also expected to enable triple-modality imaging in the future. A triplex-parameter detection method, which captures optical attenuation, optical absorption, and acoustic impedance with single-pulse excitation has already been proven in PAI.^47^ Similar to PAI, the X-ray distribution image, XACT image, and ultrasound echo image could be generated based on the corresponding signals within each pulse. Consequently, X-ray attenuation, X-ray acoustic, and ultrasonic imaging can be obtained simultaneously.

Limitations of XACT also rely on the development of X-ray generators. Due to the limited X-ray beam distribution,^48^ targets currently must be positioned sufficiently close to the X-ray source, which results in an inability to image at greater depths.

Laser-driven X-ray sources utilize intense laser beams, with nanoseconds or less pulse, couple of kHz repetition rate, and energy in the range of keV to MeV would serve as a perfect excitation source for future XACT imaging.^49^^–^^51^ This adaptability reinforces XACT’s role in elevating medical imaging practices by contributing to heightened precision and resolution.

## Data Availability

All data in support of the findings of this paper are available within the article.
